# Internal limiting membrane peeling in rhegmatogenous retinal detachment: A meta-analysis

**DOI:** 10.1371/journal.pone.0297230

**Published:** 2024-03-15

**Authors:** Guohai Chen, Radouil Tzekov, Yan Fang, Yuhua Tong, Wensheng Li

**Affiliations:** 1 Department of Ophthalmology, The Quzhou Affiliated Hospital of Wenzhou Medical University, Quzhou People’s Hospital, Quzhou, Zhejiang, PR China; 2 Department of Ophthalmology, University of South Florida, Tampa, Florida, United States of America; 3 Shanghai Aier Eye Hospital, Shanghai, PR China; 4 Shanghai Aier Eye Institute, Shanghai, PR China; 5 Aier School of Ophthalmology, Central South University, Changsha, Hunan Province, PR China; Eye Foundation Hospital / Eye Foundation Retina Institute, NIGERIA

## Abstract

**Purpose:**

To determine whether pars plana vitrectomy (PPV) with internal limiting membrane (ILM) peeling for rhegmatogenous retinal detachment (RRD) could get better functional and anatomical outcomes.

**Methods:**

A comprehensive literature search was performed to find relevant studies. A meta-analysis was conducted by comparing the weighted mean differences (WMD) in the mean change of best corrected visual acuity (BCVA) from baseline and calculating the odd ratios (OR) for rates of epiretinal membrane (ERM) formation and recurrence of retinal detachment (RD).

**Results:**

Fourteen studies were selected, including 2259 eyes (825 eyes in the ILM peeling group and 1434 eyes in the non-ILM peeling group). There was no significant difference in terms of mean change in BCVA from baseline and the rate of RD recurrence (WMD = 0.02, 95% CI, -0.20 to 0.24, P = 0.86, and OR = 0.55, 95% CI, 0.24 to 1.26, P = 0.16), but ILM peeling was associated with a significantly lower frequency of postoperative ERM formation (OR = 0.13, 95% CI, 0.06 to 0.26, P<0.00001). Similar results were obtained in a sub-analysis based on macula-off RRD.

**Conclusion:**

ILM peeling results in similar BCVA, with same rate of RD recurrence, but lower rate of postoperative ERM development. ILM peeling could be considered in selected cases with risk factors that are likely to develop an ERM.

## Introduction

Macular epiretinal membrane (ERM) formation is one of the most common postoperative complication of pars plana vitrectomy (PPV) for rhegmatogenous retinal detachment (RRD), with an incidence of 6–13% in different studies [1–3]. These membranes may cause metamorphopsia and decreased visual acuity, prompting a second PPV with membrane peeling. A relatively recent meta-analysis reported that the aggregated rate of ERM was 29% (86/295) across six studies and the rate of secondary surgery was 16% (22/141) in the eyes without primary internal limiting membrane (ILM) peeling for RRD, but without any visual outcomes [4].

ERM formation postvitrectomy is attributed to the proliferation of various cells, including cells of the retinal pigment epithelium (RPE), retinal glial cells, myofibroblasts, and hyalocytes over the ILM [1, 2]. ILM peeling during the primary surgery for RRD has been reported to prevent recurrence of ERM [5–8]. However, the visual outcomes of adjuvant ILM peeling as compared with conventional PPV without ILM peeling in RRD are controversial. While some studies report superior visual acuity in ILM-peeled eyes [9–11], some report better visual acuity in non-ILM peeled eyes [12, 13], and others report comparable vision function outcomes [6, 8, 14].

A recent meta-analysis of studies comparing ILM peeling versus no peeling during PPV for RRD found the incidence of postoperative macular ERM formation to be significantly lower in patients who had ILM peeling, but this bared no significant effect on postoperative visual acuity [15]. However, it included only one randomized controlled trial (RCT) and eight comparative studies, and analyzed macular-on and macular-off RRDs in an aggregated way. The availability of new reports prompted our decision to undertake an updated independent assessment of the problem and to undertake a meta-analysis to get more reliable results about the ILM peeing in PPV for primary RRD, to determine whether ILM peeling could get better functional and anatomical outcomes.

## Materials and methods

### Search strategy

We conducted searches of PubMed, EmBase, and ISI Web of Science, using the terms (“rhegmatogenous retinal detachment” OR “retinal detachment”) AND (“inner limiting membrane” OR “internal limiting membrane”), with the language set to English. The final search was carried out on May 28, 2023. Additional search was carried out by exploring reference lists in the originally identified articles.

### Inclusion and exclusion criteria

The criteria we applied when published studies were considered eligible for this meta-analysis were: 1. Study design: independent retrospective or prospective association study, 2. Population: participants with primary RRD without ERM detected during the initial vitrectomy, 3. Intervention: PPV with ILM peeling versus PPV without ILM peeling, and 4. Outcome variables: a) postoperative change in best-corrected visual acuity (BCVA); b) proportion of cases with ERM and recurrence retinal detachment (RD). Studies using scleral buckle were excluded. Articles reporting data from the same study, abstracts, letters to the editor, case reports, and review articles were excluded.

### Outcome measures

The functional outcome measure was mean change of post-operative BCVA expressed as logarithm of the minimal angle of resolution (logMAR) from baseline. The anatomical outcomes were evaluated by the frequency of ERM occurrence and recurrence RD.

### Data extraction

Two surgeons (G.H.C. and W.S.L.) reviewed all citations generated by the search and selected studies that matched the inclusion criteria, then extracted data from included studies. Uncertainty was resolved by discussion. The list of extracted items was as follows: first author’s name, year of publication, region, number of participants in each group, preoperative and postoperative BCVA, the proportion of patients with ERM and recurrence RD, and length of follow-up.

### Qualitative assessment

We assessed quality of studies included in this meta-analysis with a modified checklist based on the Newcastle-Ottawa Scale (NOS), in which a study was judged on three categories: selection (four items, one star each), comparability (one item, up to two stars), and exposure/outcome (three items, one star each) [16]. A nine-point scale of the NOS (range, 0–9 points) was developed for the evaluation. Studies were defined as high quality if they had more than seven points; as medium quality if they had between four and six points; and as poor quality if they had fewer than four points. Studies with NOS score above 4 points were included in the final analysis. The qualities of randomized clinical trials (RCTs) were assessed by the Jadad scale, which is a 5-point scale, assigning scores for reported randomization, masking and participant withdrawals [17]. Studies scoring less than 3 points were excluded from this study.

### Statistical analysis

The meta-analysis was conducted by using Cochrane Review Manager (RevMan, software version 5.1, Copenhagen, Denmark: The Nordic Cochrane Center, The Cochrane Collaboration, 2011). When analyzing continuous variables, the weighted mean difference (WMD) was calculated, while the odds ratios (OR) were obtained for dichotomous variables (e.g., number of eyes) and a 95% confidence interval (CI) was reported. P<0.05 was considered statistically significant on the test for overall effect. The I^2^ statistic was calculated to assess heterogeneity between studies (I^2^>50% or P<0.05 was considered representative of significant statistical heterogeneity) [18]. If the I^2^ statistic test turned out as statistically significant, a random-effects model was used. In case where I^2^ statistic was not significant a fixed-effects model was applied. For determining the weight of effect size in a study we used the inverse variance method, generally accepted as suitable for use in meta-analysis [19]. The level of bias in the selected publications was assessed by Begg’s rank correlation test and by Egger’s linear regression test with P<0.05 considered significant [20, 21].

## Results

### Overall characteristics of selected trials and quality assessment

**Fig 1:**
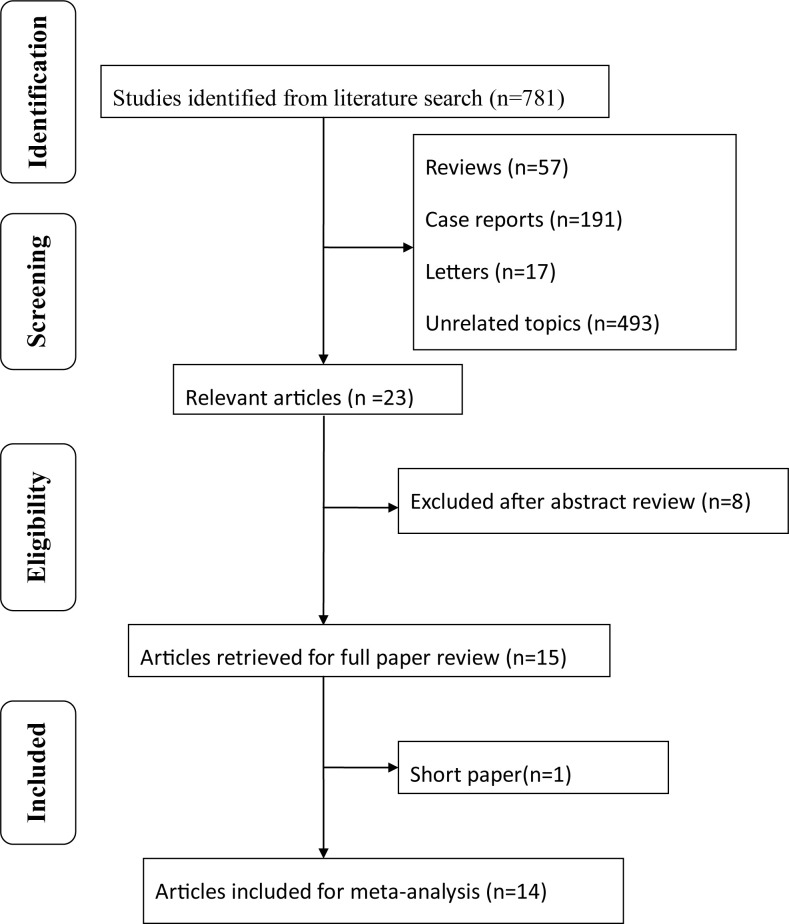
Flow diagram of studies included in this meta-analysis.

The search resulted in 781 entries being identified. Of these, 766 did not meet the inclusion criteria listed above and were rejected. One study was rejected as being a short paper, its quality could not be assessed [22]. This resulted in fourteen studies remaining which were included in this meta-analysis [5–14, 23–26]. Fig 1 depicts the overall study selection process. Most of the studies were retrospective, except two, which were prospective [5, 25], and two being RCTs [12, 24]. Outcome data were available from 2259 eyes of 2259 participants, comprising 825 eyes in the ILM peeling group and 1434 eyes in the non-ILM peeling group, respectively. All the nonrandomized studies had high quality scores of 7 according to the NOS, and the RCT fulfilled the quality criteria (3 points), according to the Jadad scale. Seven studies used brilliant blue G (BBG) for ILM staining [5, 9, 12, 14, 23, 24, 26], while two studies used indocyanine green (ICG) [11, 24], two studies used trypan blue (TB) [7, 10], one study reported results of with triamcinolone acetonide-assisted (TA) ILM peeling [6], one study–BBG or TB [8], and one study–BBG, TB, ICG or TA [13]. Nine studies included only macula-off RRD [5, 7, 10, 12, 14, 23–26], and five studies included both macula-on and macula-off RRD [6, 8, 9, 11, 13].

The proliferative vitreoretinopathy (PVR) stage was graded as defined by the classification methods of the Retina Society Terminology Committee [27]. Most studies included patients with PVR grade ≤ C1, only one study had patients with PVR grade of <D2 [7]. Six studies used expansile gas tamponade [8, 10, 11, 14, 23, 24], while four studies used silicon oil tamponade [5, 7, 12, 25], and four studies used expansile gas or silicon oil tamponade [6, 9, 13, 26]. In most studies the removal of silicon oil was at 3 months, expect one study with silicon oil being in situ during 6 months follow up period [25]. Table 1 provides the characteristics of the included studies.

**Table 1:** Characteristics of included studies.

Study group (year)	Location	Design	Macula status	PVR grade	ILM staining	Tamponade	Treatment	No. eyes	Age (year)	Baseline BCVA (LogMAR)	Final BCVA (LogMAR)	Follow-up (months)
Abdullah 2020	Egypt	Prospective	Macula off	<C1	BBG	SO	ILM peeling	30	49.9±13.1	1.9±0.3	0.9±0.15	6
							No ILM peeling	30	44.6±11.2	1.9±0.5	0.6±0.2	6
Akiyama 2016	Japan	Retrospective	Macula on and off	A	TA	Air, SF6, C3F8 or SO	ILM peeling	58	58.2±12.1	0.44±0.75	0.08±0.27	6
							No ILM peeling	44	58.5±10.6	0.63±0.81	0.03±0.19	6
Aras 2009	Turkey	Retrospective	Macula off	<D2	TB	SO	ILM peeling	20	52.7±12.6	1.56±0.54	0.60±0.30	24.6
							No ILM peeling	22	52.2±13.3	1.86±0.38	0.72±0.35	34.1
Arias 2020	Spain	Retrospective	Macula on and off	<C1	BBG and TB	SF6 or C3F8	ILM peeling	70	60.2±12.5	2	0.1	15.9
							No ILM peeling	70	60.5±12.4	1	0	48.8
Blanco-Teigeiro 2018	Spain	Retrospective	Macula off	A	BBG	SF6	ILM peeling	30	67±9.8	1.9±0.52	0.4±0.25	16
							No ILM peeling	32	65±14.8	1.15±0.52	0.3±0.33	20
Eissa 2018	Egypt	RCT	Macula off	<C1	BBG	SO	ILM peeling	20	52.7±10.3	1.9±0.4	1.0±0.4	6
							No ILM peeling	23	47.7±15.0	1.9±0.2	0.4±0.4	6
Forlini 2017	Italy	Retrospective	Macula on and off	<C2	BBG	SF6, C3F8 or SO	ILM peeling	78	62.2±10.5	1.19±0.7	0.48±0.4	12
							No ILM peeling	81	63.4±11.8	1.24±0.7	0.77±0.6	12
Foveau 2018	France	Retrospective	Macula off	B	BBG	SF6	ILM peeling	37	64.4±9.3	1.81±0.56	0.41±0.4	6
							No ILM peeling	38	65.3±9.2	1.78±0.54	0.43±0.22	6
Garweg 2018	Switzerland	Retrospective	Macula off	<C2	TB	SF6	ILM peeling	61	NA	1.48	NA	6
							No ILM peeling	28	NA	1.42	NA	6
Kumar 2020	India	RCT	Macula off	≤C1	BBG	SF6	ILM peeling	30	46.23±14.3	1.87±0.48	0.68±0.48	15.5
							No ILM peeling	30	43.86±12.3	1.53±0.75	0.59±0.46	14
Mahmood 2021	Pakistan	Prospective	Macula off	B and C	ICG	SO	ILM peeling	26	51.9±10.79	1.79±0.26	0.75±0.32	6
							No ILM peeling	30	54.2±7.75	1.73±0.29	0.74±0.33	6
Nam 2015	Korea	Retrospective	Macula on and off	A	ICG	SF6 or C3F8	ILM peeling	70	48.2±17.8	0.76±0.84	NA	12
							No ILM peeling	65	47.9±19.0	0.85±0.87	NA	12
Obata 2021	Japan	Retrospective	Macula on and off	A	TA,BBG,ICG or TB	Air, SF6, C3F8 or SO	ILM peeling	142	59.3±8.6	0.47±0.69	NA	6
							No ILM peeling	745	58.5±9.4	0.55±0.8	NA	6
Sousa 2020	Portugal	Retrospective	Macula off	<C3	BBG	SF6, C2F6, C3F8 or SO	ILM peeling	153	63.2±12.5	2±0.5	NA	12
							No ILM peeling	196	61.7±14.2	2±0.43	NA	12

Abbreviations: PVR, proliferative vitreoretinopathy; ILM, internal limiting membrane; BCVA, best corrected visual acuity; LogMAR, logarithm of the minimal angle of resolution; RCT, randomized clinical trials; BBG, brilliant blue G; TA, triamcinolone acetonide; TB, trypan blue; ICG, indocyanine green; SO, silicone oil; SF_6_, sulfur hexafuoride; C_3_F_8_, octafuoropropane; NA, not available.

### Best corrected visual acuity

**Fig 2:**
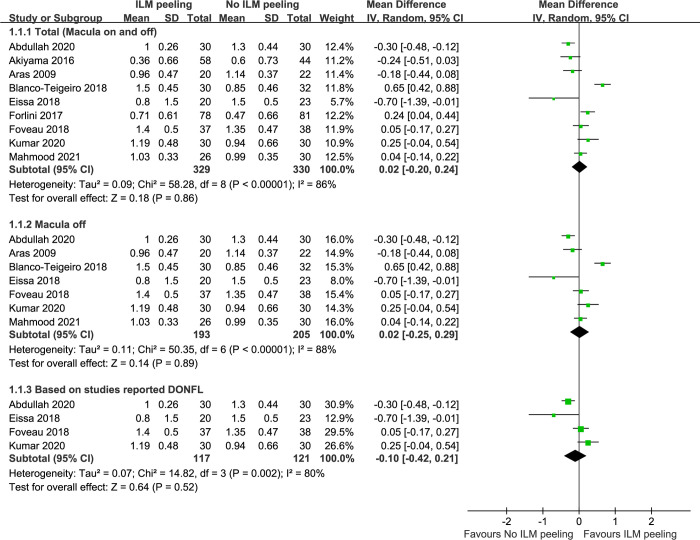
The mean change in best corrected visual acuity (logMAR units) from baseline between vitrectomy with ILM peeling vs. vitrectomy without ILM peeling. ILM, internal limiting membrane; SD, standard deviation; IV, inverse variance; CI, confidence interval; DONEL, dissociated optic nerve fiber layer.

Nine studies involving 659 eyes compared ILM peeling to non-ILM peeling in terms of mean change in logMAR BCVA from baseline. The combined results showed that both groups showed improvement in BCVA (+0.91 logMAR in the ILM peeling group and +0.91 logMAR in the non-ILM peeling group). Overall, there was no significant difference between ILM peeling and non-ILM peeling in terms of mean change of logMAR BCVA post operation (WMD = 0.02, 95% CI, -0.20 to 0.24, P = 0.86), but with heterogeneity identified (Fig 2). In a subgroup analysis including only macula-off RRD, similarly to the main analysis, heterogeneity was identified and a random-effects model was applied to the data, showing no significant difference in BCVA between ILM peeling and non-ILM peeling (WMD = 0.02, 95% CI, -0.25 to 0.29, P = 0.89) (Fig 2). Five studies reported dissociated optic nerve fiber layer (DONFL) appearance after surgery [5, 8, 12, 23, 24]. Overall, a total of 92 out of 187 (49.2%) eyes developed DONFL in the ILM peeling group, while no eye (0/191) developed DONFL in the non-ILM peeling group. There was no significant difference in BCVA between ILM peeling and non-ILM peeling in the sub-analysis of these studies reported DONFL (WMD = 0.10, 95% CI, -0.42 to 0.21, P = 0.52) (Fig 2). A subgroup analysis based on type of the tamponade used, showed no significant difference in BCVA between ILM peeling and non-ILM peeling cases for the silicon oil subgroup or the expansile gas tamponade subgroup (WMD = -0.19, 95% CI, -0.42 to 0.03, P = 0.1 and WMD = 0.32, 95% CI, -0.06 to 0.69, P = 0.1, respectively) (S1 Fig). Begg’s test and Egger’s test indicated no statistically significant evidence of publication bias.

### Postoperative ERM formation

**Fig 3:**
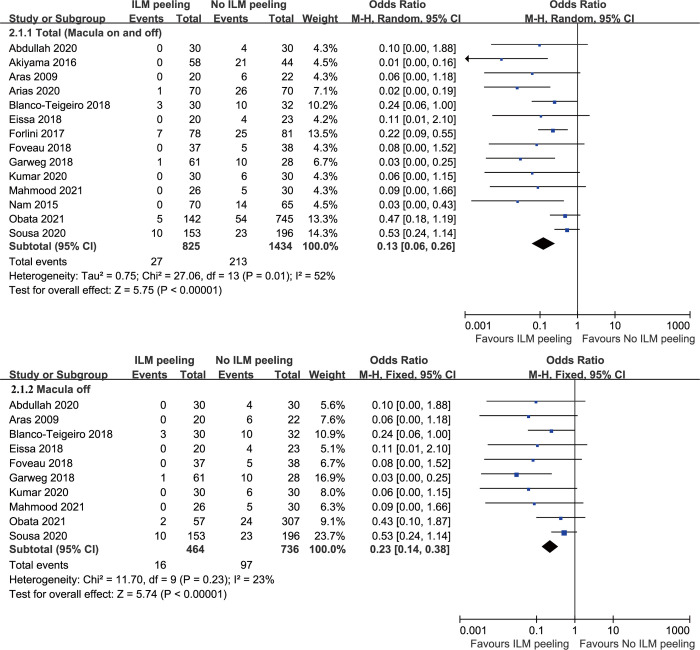
The incidence of macular epiretinal membrane formation between vitrectomy with ILM peeling vs. vitrectomy without ILM peeling. ILM, internal limiting membrane; M-H, Mantel-Haenszel; CI, confidence interval.

Fourteen studies involving 2259 eyes compared ILM peeling to non-ILM peeling in terms of postoperative ERM. ERM developed in 3.27% (27/825) of the eyes in the ILM peeling group, and 14.85% (213/1434) of the eyes in the non-ILM peeling group. The ILM peeling group was associated with a significantly lower frequency of ERM formation compared to the non-ILM peeling group (OR = 0.13, 95% CI, 0.06 to 0.26, P<0.0001), with heterogeneity identified and a random-effects model applied to the data (Fig 3). In a subgroup analysis that included only macula-off RRD, similarly to the main analysis, ILM peeling was associated with a significantly lower rate of ERM formation (OR = 0.23, 95% CI, 0.14 to 0.38, P<0.0001), with no heterogeneity identified and a fixed-effects model applied to the data (Fig 3). In a subgroup analysis based on type of the tamponade used, ILM peeling was also associated with a significantly lower rate of ERM formation in both silicon oil and expansile gas tamponade subgroups (OR = 0.08, 95% CI, 0.02 to 0.34, P = 0.0007 and OR = 0.06, 95% CI, 0.03 to 0.13, P<0.0001, respectively) (S2 Fig). Begg’s test and Egger’s test indicated no statistically significant evidence of publication bias.

### Recurrence of retinal detachment

**Fig 4:**
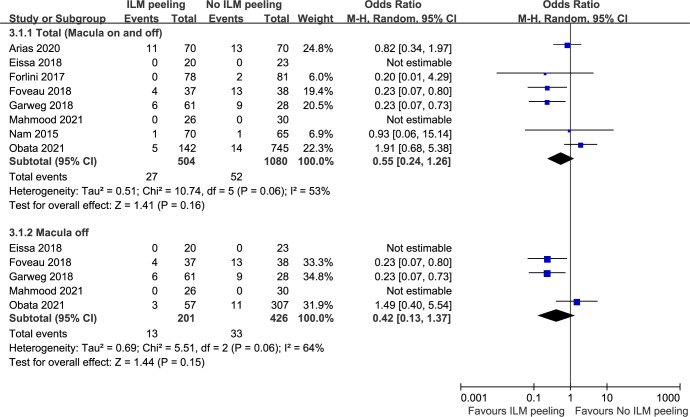
The incidence of retinal detachment recurrence between vitrectomy with ILM peeling vs. vitrectomy without ILM peeling. ILM, internal limiting membrane; M-H, Mantel-Haenszel; CI, confidence interval.

Eight studies involving 1584 eyes compared ILM peeling to non-ILM peeling in terms of the recurrence of RD. Overall, a total of 27 out of 504 (5.36%) eyes had recurrence of RD in the ILM peeling group, and 52 out of 1080 (4.81%) eyes in the non-ILM peeling group. There was no significant difference in the rate of RD recurrence between ILM peeling and non-ILM peeling (OR = 0.55, 95% CI, 0.24 to 1.26, P = 0.16), with heterogeneity identified and a random-effects model applied to the data (Fig 4). Subgroup analysis included only macula-off RD and showed results similar to the result from the main analysis (OR = 0.42, 95% CI, 0.13 to 1.37, P = 0.15) (Fig 4). Begg’s test and Egger’s test indicated no statistically significant evidence of publication bias.

## Discussion

In this meta-analysis, we reviewed fourteen studies, including 2259 eyes (825 eyes in the ILM peeling group and 1434 eyes in the non-ILM peeling group). In terms of rate of postoperative ERM formation, the result of our meta-analysis was in favor of ILM peeling (3.27% VS. 14.85%). In assessing the mean change in logMAR BCVA from baseline, both groups gained similar improvement in BCVA (+0.91 logMAR vs. +0.91 logMAR), and the rate of RD recurrence was also similar (5.36% vs. 4.81%).

Regamtogenous retinal detachment is generally associated with the presence of posterior vitreous detachment (PVD) [14]. However, even if a complete PVD occurred, some cortical vitreous was frequently observed during PPV for RRD, at the rate of approximately 60–75% [28–30]. ERM development after RRD repair is mostly attributed to the RPE cells and other progenitor cells that migrate through retinal breaks and the presence of a residual vitreous cortex over the retinal surface [31]. ILM peeling ensures complete removal of the posterior vitreous cortex and progenitor cells from the scaffold they need to proliferate on the macular surface [32, 33]. Our meta-analysis indicates that ILM peeling was associated with an approximately 4.5-fold lower frequency of postoperative ERM formation (27/825 vs. 213/1434), supporting the conclusion that ILM peeling prevents ERM formation after PPV for RRD. One study found that macular ILM peeling prevents macular ERM formation. However, the ERM did develop outside the peeled area where the ILM scaffold was still persisting [24]. This finding strengthens the ‘‘ILM scaffold” concept for ERM formation, suggesting an ILM peeling in the entire macular area (2-disc diameters all around the fovea) will be more beneficial than a smaller-sized peeling [24].

The possible adverse events of ILM peeling, such as a ‘‘DONFL appearance” or ‘‘inner retinal dimpling” have been discussed [34]. In our meta-analysis, there were 92/187 eyes developed DONFL in the ILM peeling group and without DONFL in the non-ILM peeling group, but their number did not appear to correlate significantly with final BCVA in the sub-analysis of these studies. Several investigations also have reported that DONFL or retinal dimples do not result in deterioration of VA, visual fields, or retinal sensitivity [35–38]. It is plausible that with lower rate of ERM in the ILM peeling group, better BCVA could be observed. However, our analysis showed no difference between cases with ILM peeling vs. cases without ILM peeling in terms of mean change in logMAR BCVA postoperatively. It is worth noting that in this meta-analysis, most studies were conducted with a short follow-up period (6–12 months). Therefore, it is possible that ERM may become thicker over a longer period of time and lead to outer retinal distortion, and then the visual acuity outcome difference may reach significance. Thus, follow-up studies using longer follow-up period would help to determine the longitudinal visual prognosis of ILM peeling in RRD repair.

In terms of the recurrence of RD, eight studies reported the data on RD recurrence, and only two studies found that ILM peeling with a lower rate of RD recurrence in the ILM peeling group. Foveau et al reasoned that the absence of ILM at the macula may reduce retinal tension transmitted to the posterior pole by peripheral or vitreous base contraction and may also increase macular compliance in the presence of peripheral contraction, which may explain the lower redetachment rate in ILM peeling group in their study [23]. However, in our meta-analysis, the rate of RD recurrence was similar between ILM peeling and non-ILM peeling at ~5% (27/504 vs. 52/1080). Presumably the tractional forces on ILM from macular ERM would be most significant at the macula, with less peripheral traction, which may be the reason why non-ILM peeling with more ERM formation was with similar redetachment rate. The relationship between ILM peeling and RD recurrence rate may need RCTs with a larger sample size to provide more definitive information.

Evaluation of the visual prognosis proved that ILM peeling did not cause a reduction in the final BCVA. However, studies using microperimetry and focal macular electroretinogram have reported reduced retinal sensitivity and pathologic changes in the Müller cells that were associated with ILM peeling [5, 39–41]. Thus, it appears that ILM peeling is preferable to be considered and performed in selected cases that are likely to develop an ERM. Risk factors for post-PPV ERM development include multiple, large or posterior retinal breaks, vitreous hemorrhages, as well as macular detachment of longer duration [1, 2, 42]. One study found that the wrinkling on the retinal surface evaluated presurgically on enface optical coherence tomographic images is an early sign of postsurgical ERM growth, which could be an indicator for ILM peeling during vitrectomy for RRDs [43].

This work carries some limitations which should be acknowledged. First, most included studies were carried out with small sample size and were retrospective, which may affect the results. Thus, prospective or randomized clinical trials with larger sample size are needed to provide more definitive information. Second, ICG was used in some studies, which could be bound to ILM for a long period of time and may be toxic to the surrounding tissue [44]. Third, most studies included only macula-off RRD [5, 7, 10, 12, 14, 23–26], and others included both macula-on and macula-off RRD [6, 9, 11, 13], without reporting the data of only macula-on RRD. Thus, additional studies focusing on ILM peeling in macula-on RRD would be helpful to assess the efficacy and safety in this condition. Fourth, most studies included patients with PVR grade ≤C1, only one study with PVR grade <D2 [7]. Therefore, the results of complex RDs are underrepresented in this analysis and will be subject to future studies. Another limitation is that most studies were with a short follow-up (6 months) [5, 6, 10, 12, 13, 23, 25]. The minimum follow-up of 6 months may have been insufficient to detect any difference in BCVA between the two groups. A recent meta-analysis study, which compared clinical outcomes of ILM peeling versus non-ILM peeling during PPV for idiopathic ERM, demonstrated significantly better BCVA in the non-ILM peeling group at the 12 months, whereas the patients in the ILM peeling group had significantly better postoperative BCVA after 18 months [45].

In summary, the present meta-analysis confirmed that ILM peeling results in similar BCVA, with same rate of RD recurrence, but lower rate of postoperative ERM development. ILM peeling could be considered in selected cases with risk factors that are likely to develop an ERM.

## Supporting information

S1 Fig The mean change in best corrected visual acuity (logMAR units) from baseline between vitrectomy with ILM peeling vs. vitrectomy without ILM peeling based on type of the tamponade used. (EPS)

S2 Fig The incidence of macular epiretinal membrane formation between vitrectomy with ILM peeling vs. vitrectomy without ILM peeling based on type of the tamponade used. (EPS)

S1 Checklist PRISMA checklist. (DOC)
